# Identification of Novel Avian Influenza Virus Derived CD8+ T-Cell Epitopes

**DOI:** 10.1371/journal.pone.0031953

**Published:** 2012-02-23

**Authors:** Sylvia S. N. Reemers, Daphne A. van Haarlem, Alice J. A. M. Sijts, Lonneke Vervelde, Christine A. Jansen

**Affiliations:** Department of Infectious Diseases and Immunology, Faculty of Veterinary Medicine, Utrecht University, Utrecht, the Netherlands; University of Montreal, Canada

## Abstract

Avian influenza virus (AIV) infection is a continuing threat to both humans and poultry. Influenza virus specific CD8+ T cells are associated with protection against homologous and heterologous influenza strains. In contrast to what has been described for humans and mice, knowledge on epitope-specific CD8+ T cells in chickens is limited. Therefore, we set out to identify AIV-specific CD8+ T-cell epitopes. Epitope predictions based on anchor residues resulted in 33 candidate epitopes. MHC I inbred chickens were infected with a low pathogenic AIV strain and sacrificed at 5, 7, 10 and 14 days post infection (dpi). Lymphocytes isolated from lung, spleen and blood were stimulated ex vivo with AIV-specific pooled or individual peptides and the production of IFNγ was determined by ELIspot. This resulted in the identification of 12 MHC B12-restricted, 3 B4-restricted and 1 B19-restricted AIV- specific CD8+ T-cell epitopes. In conclusion, we have identified novel AIV-derived CD8+ T-cell epitopes for several inbred chicken strains. This knowledge can be used to study the role of CD8+ T cells against AIV infection in a natural host for influenza, and may be important for vaccine development.

## Introduction

Influenza A virus infections affect both humans and poultry. Seasonal influenza infections affect millions of humans worldwide each year and outbreaks of avian influenza viruses (AIV) including the highly pathogenic H5N1 viruses in wild birds and poultry occur regularly [1], [2]. Furthermore, AIV are able to infect humans [3]–[5] which makes these zoönotic viruses a significant threat for human health because of their pandemic potential.

It is well established that the humoral immune response plays an important role in controlling influenza virus infections [6]–[9], and the induction of neutralizing antibodies is nowadays one of main criteria to determine vaccine efficacy [10]. Antibodies are mainly directed against the highly variable surface proteins haemagglutinin (HA) and neuraminidase (NA) which continuously change under “antigenic drift”, and viruses escape from recognition by virus-specific antibodies. Under these circumstances the induction of cross-protective cytotoxic CD8+ T cells that recognize conserved epitopes may be important [11].

Studies in humans and mice have shown that influenza-specific CD8+ T cells are involved in protection against influenza virus infection [12]–[14]. CD8+ T-cell responses are mainly directed against conserved proteins like the nucleoprotein (NP) and matrix 1 (M1) protein [15], [16] and have been shown to provide cross-protection against heterologous influenza strains [17]–[19].

Also in chickens, which are a natural host for AIV, CD8+ T cells are associated with protection; immunization with low pathogenic AIV (LPAIV) of the H9N2 type results in protection against a highly pathogenic H5N1 AIV (HPAIV) [20], [21]. Cross-reactivity between CD8+ T cells specific for seasonal influenza and H5N1 HPAIV has been described [22] as well as cross-reactivity between LPAIV of the H9N2 and H7N2 type [23]. Furthermore, conserved epitopes have been detected in influenza viruses isolated from humans and avian species [24]. Taken together, these data show that influenza-specific CD8+ T cells exist in chickens and are associated with protection against homologous and heterologous influenza strains.

In contrast to what has been described for humans and mice, knowledge on influenza epitope-specific CD8+ T cells in chickens is limited. Cross-reactive T-cell responses to the AIV proteins HA and NP have been reported in chickens inoculated with plasmids expressing viral proteins HA and NP [25] or non-replicating adenovirus vectors expressing these proteins [26]. However, AIV-derived epitopes recognized by these CD8+ T cells are still unknown.

The chicken MHC, also called “B locus”, is more compact and differently organised than the mammalian MHC. The B-F/B-L region within the B locus contains the classical class I and class II^β^ chains, and determines allograft rejection, strong mixed lymphocyte reactions and the cellular control of antibody production [27]–[32]. For a number of common chicken MHC haplotypes, MHC class I restricted peptide motifs have been determined. Anchor residues involved in binding to the MHC class I molecules of these different haplotypes were found to be just as critical as to what has been described for mammalian MHC class I [32]–[34].

In this study, we set out to identify novel AIV-specific CD8+ T-cell epitopes. To this end, epitopes in the viral proteins NP and M1 were predicted based on anchor residues described for MHC B4, B12, B15, B19 and B21. Screening of these peptides resulted in the identification of 16 novel AIV-specific CD8+ T-cell epitopes; 12 B12-restricted epitopes, 3 B4-restricted epitopes and 1 B19-restricted epitope.

## Results

### Analysis of T-cell frequencies upon LPAIV infection

**Figure 1:**
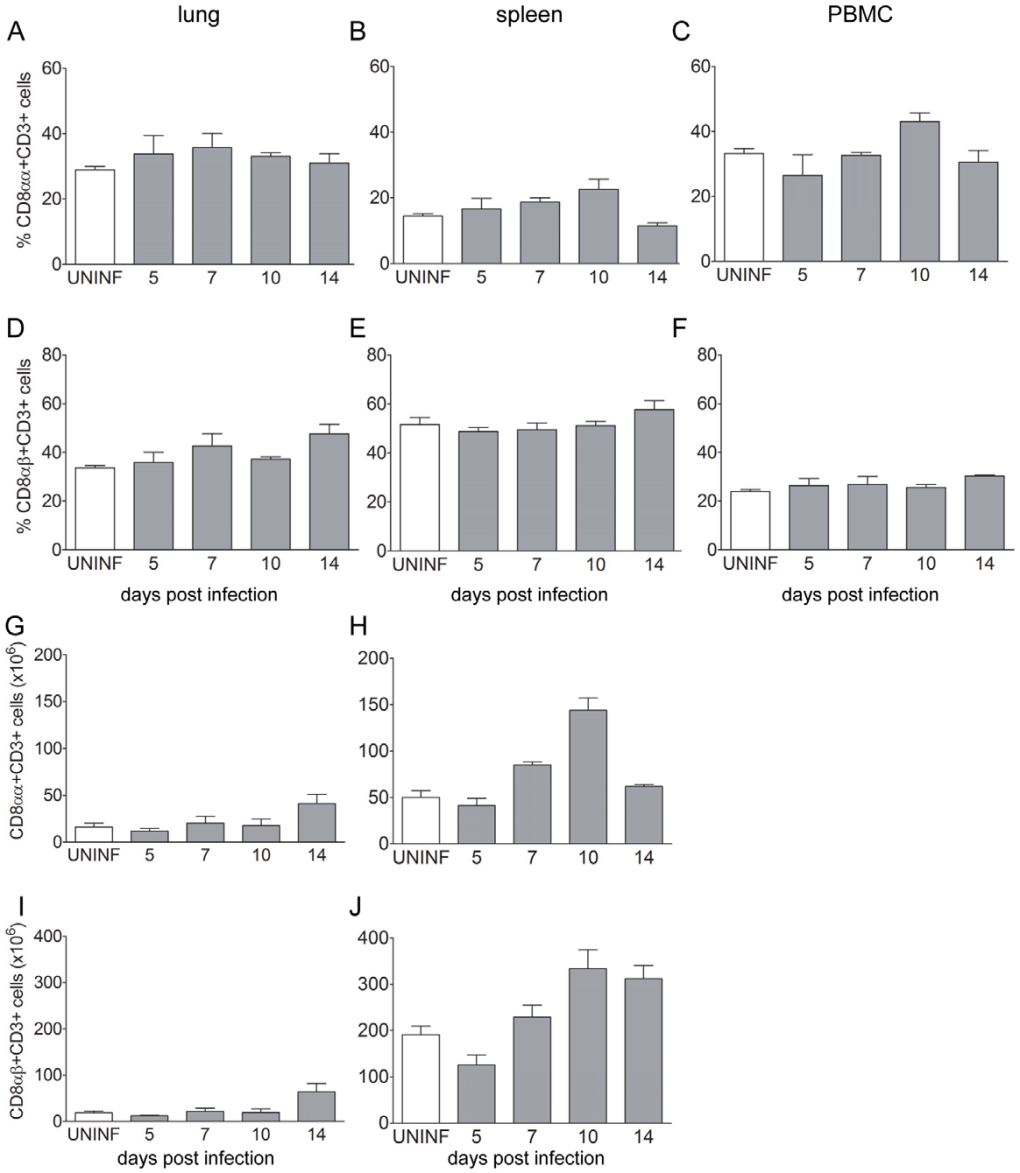
CD8+ T-cell frequencies in tissues of LPAIV infected chickens. Percentages of CD8αα+CD3+ T cells (A–C) and CD8βα+CD3+ T cells (D–F) were analysed by flowcytometry in lung, spleen and PBMC at several days post infection. Absolute numbers were calculated by multiplying the percentage of CD8αα+CD3+ T or CD8βα+CD3+ T cells with the total number of cells isolated from lung (G, I) and spleen (H, J). Mean plus SEM is shown. In white: uninfected controls (UNINF, n = 12), in grey infected birds (n = 3 per time point).

To investigate if infection with LPAIV would result in an influx of T cells into the lung, we determined the frequencies of different T-cell subsets by flowcytometry. No differences in the percentage of CD8αα+ T cells in the lungs was observed in infected birds compared to uninfected controls (Fig. 1A) while the number of CD8αα+ T cells in lung did increase upon infection (Fig. 1G), Interestingly, at 10 dpi frequencies of CD8αα+ T cells were slightly higher in infected birds compared to uninfected controls in both spleen (14.4±0.8% versus 22.5±3.0%, Fig. 1B) and PBMC (33.1±1.5% versus 43.1±2.7%; Fig. 1C). This increase in splenic CD8αα+ T cells was more pronounced when the number of cells was analysed (50.0±7.1×10^6^ versus 143.6±13.7×10^6^, Fig. 1H). At 14 dpi, the percentage of CD8αβ+ T cells was slightly increased compared to uninfected controls in lung (47.6±3.9% versus 33.7±1.0%; Fig. 1D), spleen (51.9±2.9% versus 57.6±3.6%; Fig. 1E) and PBMC (30.3±0.5 versus 24.0±0.8%; Fig. 1F). Again, differences in numbers of CD8αβ+ T cells between infected birds and uninfected controls were much higher in both lung (18.3±4.1×10^6^ versus 64.3±17.8×10^6^; Fig. 1I) and spleen (191.1±17.8×10^6^ versus 333.9±40.2×10^6^; Fig. 1J). Similar results were observed for numbers of CD4+ T cells and γδ+ T cells (data not shown). Thus, infection with LPAIV results in increased numbers of CD8+ T-cells.

### Identification of AIV-specific CD8+ T-cell epitopes using peptide pools

Avian influenza virus-specific IFNγ-producing CD8+ T cells were analysed in the lung, which is a target organ of low pathogenic avian influenza viruses. ELIspot was performed upon stimulation of lung cells with pools of peptides that were derived from the avian influenza viral proteins NP and M1 and were selected based on the presence of MHC binding motifs (Tables 1 and 2, Tables S1 and S2).

**Table 1:** Epitope prediction for MHC B12 based on anchor residues.

	B12: X-X-X-X-V/I-X-X-(X)-V/L/I		
peptide	sequence	MHC restriction	Viral protein
A1	NATE**I**RAS**V**	B12	nucleoprotein
A2	IRAS**V**ERM**V**	B12	nucleoprotein
A3	EGRL**I**QNS**I**	B12	nucleoprotein
A4	NSIT**I**ERM**V**	B12	nucleoprotein
A5	DGKW**V**REL**I**	B12	nucleoprotein
A6	AVKG**V**GTM**V**	B12	nucleoprotein
A7	VGTM**V**MEL**I**	B12	nucleoprotein
A8	LIRM**I**KRG**V**	B12	nucleoprotein
A9	GNAE**I**EDL**I**	B12	nucleoprotein
A10	QNSQ**V**FSL**I**	B12	nucleoprotein
A11	EDLR**V**SSF**I**	B12	nucleoprotein
A12	PTFS**V**QRN**L**	B12	nucleoprotein
B1	VERM**V**GG**I**	B12	nucleoprotein
B2	DGKW**V**RE**L**	B12	nucleoprotein
B3	DKEE**I**RR**I**	B12	nucleoprotein
B4	AGAA**V**KG**V**	B12	nucleoprotein
B5	VGTM**V**ME**L**	B12	nucleoprotein
B6	VMEL**I**RM**I**	B12	nucleoprotein
B7	GNAE**I**ED**L**	B12	nucleoprotein
B8	LPAC**V**YG**L**	B12	nucleoprotein
B9	QNSQ**V**FS**L**	B12	nucleoprotein
B10	QGRG**V**FE**L**	B12	nucleoprotein
F1	VETY**V**LS**I**	B12	matrix protein 1
F2	ILGF**V**FT**L**	B12	matrix protein 1
F3	KDDL**I**EN**L**	B12	matrix protein 1
F4	LLTE**V**ETY**V**	B12	matrix protein 1
F5	VETY**V**LSI**V**	B12	matrix protein 1
F6	LKAE**I**AQR**L**	B12	matrix protein 1
F7	KTRP**I**LSP**L**	B12	matrix protein 1
F8	LTKG**I**LGF**V**	B12	matrix protein 1
F9	RRRF**V**QNA**L**	B12	matrix protein 1
F10	RMGT**V**TTE**V**	B12	matrix protein 1
F11	VTTE**V**AFG**L**	B12	matrix protein 1

Predicted epitopes and their localization based on anchor residues that have been described for B12 [32], [33]. X represents any amino acid. Anchor residues specific for the different MHC types are indicated in bold. A variable number of amino acids between the anchor residues is indicted with (X).

**Table 2:** Epitope prediction for MHC B4, B15, B19 and B21 based on anchor residues.

	B4: X-**D/E**-X-X-**D/E**-X-X-(X)-**E/L/I**		
A1	Y**E**QM**E**TG**E**	B4	nucleoprotein
A2	Y**D**KE**E**IRR**I**	B4	nucleoprotein
A3	A**E**IE**D**LIF**L**	B4	nucleoprotein
A4	M**E**TM**D**SST**L**	B4	nucleoprotein
	B15: X-**R**-X-X-X-X-X-(X)-**Y**		
A5	G**R**RTRIA**Y**	B15	nucleoprotein
	B19: X-**R**-X-X-X-X-X-**Y/P/L/F**		
A6	E**R**MVLSA**F**	B19	nucleoprotein
A7	K**R**GVNDR**N**F	B19	nucleoprotein
A8	G**R**RTRIA**Y**	B19	nucleoprotein
A9	I**R**GTRVV**P**	B19	nucleoprotein
A10	T**R**VVPRG**Q**L	B19	nucleoprotein
A11	E**R**ATIMA**A**F	B19	nucleoprotein
A12	I**R**MMESA**R**P	B19	nucleoprotein
B1	A**R**PEDVS**F**	B19	nucleoprotein
B2	T**R**PILSP**L**	B19	matrixprotein 1
B3	E**R**GLQRR**R**F	B19	matrixprotein 1
B4	R**R**RFVQN**A**L	B19	matrixprotein 1
B5	R**R**FVQNA**L**	B19	matrixprotein 1
B6	I**R**HENRM**V**L	B19	matrixprotein 1
B7	M**R**TIGTH**P**	B19	matrixprotein 1
B21: X-**H/K/R**-X-X-X-X-X-(X)-**E/D**-X-**A/V/L/I/F/M**			
B8	R**R**DGKWVR**E**LI	B21	nucleoprotein
B9	C**H**SAAFE**D**LRV	B21	nucleoprotein
B10	G**R**TSDMRT**E**II	B21	nucleoprotein
B11	W**R**QANNGE**D**A	B21	nucleoprotein
B12	N**R**MGTVTT**E**VA	B21	matrixprotein 1
C1	L**K**DDLIEN**L**QA	B21	matrixprotein 1

Predicted epitopes and their localization based on anchor residues that have been described for B4, B15, B19 and B21 [32]–[34]. X represents any amino acid. Anchor residues specific for the different MHC types are indicated in bold. A variable number of amino acids between the anchor residues is indicted with (X).

**Figure 2:**
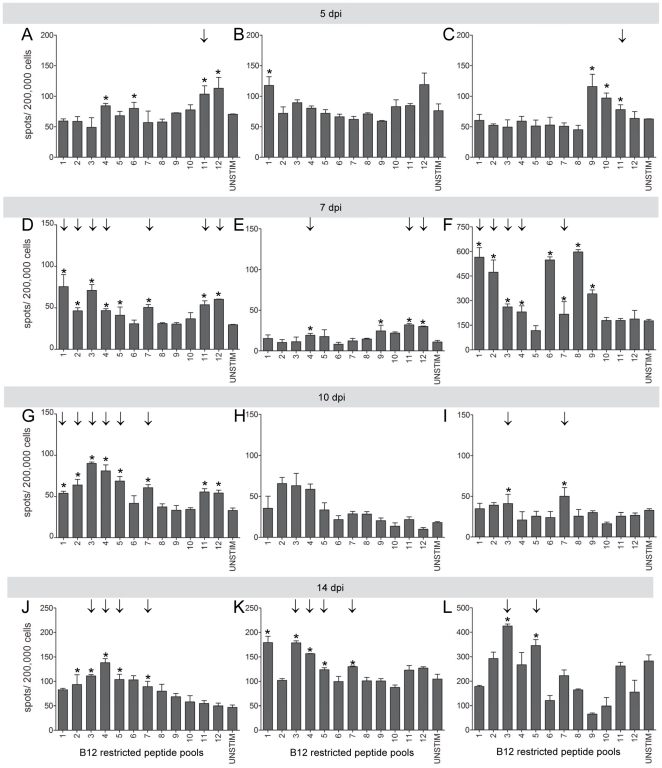
Identification of MHC B12-restricted CD8+ T-cell epitopes using peptide pools. Lung cells were stimulated with B12-restricted peptide pools and IFNγ-producing cells were determined by IFNγ ELIspot analysis. Results for three individuals birds are shown at 5 dpi (A–C), 7 dpi (D–F), 10 dpi (G–I) and 14 dpi (J–L). Mean plus SEM is shown, n = 3 per group. Positive responses (*) and “significant” peptides inducing a positive response in 2 out of 3 chickens (↓) are indicated.

Since most of the predicted MHC binders were B12-restricted, a kinetic study was performed in which IFNγ-producing AIV-specific CD8+ T cells were analysed at 5, 7, 10 and 14 dpi in H7N1 infected B12 chickens. For a representative image of positive and negative ELIspot wells, see Figure S1A. At 5 dpi, a number of peptide pools triggered IFNγ production that was higher than in the unstimulated control. Based on the strict criteria we used in order to focus only on epitopes that elicit responses in a majority of individuals only pool 11 induced a significantly positive response which was defined as the mean of the triplicates is higher than the mean of the triplicates plus 2 times the standard error of unstimulated control and positive in more than 2 birds (Fig. 2A–C). As is shown in Figure 2D–F, at 7 dpi the number of pools inducing significant IFNγ production had strongly increased which resulted in 7 significantly positive pools. Also the number of spots had increased compared to 5 dpi. At 10 dpi again 7 pools tested significantly positive (Fig. 2G–I). Most pools were significantly positive both at 7 dpi and 10 dp, with the exception of pool 12 (only significantly positive at 7 dpi) and pool 5 (only significantly positive at 10 dpi). At 14 dpi, both the number of pools that induced IFNγ-producing cells as well as the number of significantly positive pools had decreased compared to 7 and 10 dpi (Fig. 2J–L). Three of the B12-restricted peptide pools that were significantly positive in the lung were also found positive in PBMC and spleen (pool 1, 3, 7; data not shown).

**Figure 3:**
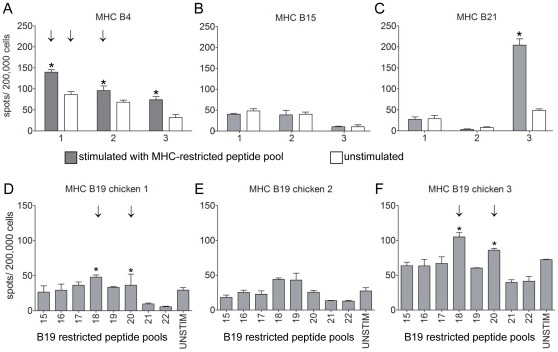
Identification of MHC B4-, B15-, B19- and B21-restricted CD8+ T-cell epitopes using peptide pools. Lung cells isolated at 10 dpi were stimulated with B4 restricted peptide pools (A) or, B15 (B), B21 (C) and B19-restricted peptide pools (D–F) and IFNγ producing cells were determined by IFNγ Elispot analysis. Mean plus SEM is shown, n = 3 per group. Positive responses (*) and “significant” peptides inducing a positive response in 2 out of 3 chickens (↓) are indicated.

Testing of MHC-B4 restricted peptides in the lung of H7N1 infected B4 chickens at 10 dpi resulted in 1 significantly positive peptide pool (Fig. 3A). No response was observed in lung cells of infected B15 chickens upon stimulation with a peptide predicted to bind MHC B15 (Fig. 3B). The pool of potential B21 binders induced IFNγ-producing cells in 1 out of 3 infected B21 chickens (Fig. 3C). Testing of MHC-B19 restricted peptides resulted in 2 significantly positive peptide pools in lung (Fig. 3D–F) which was elicited by 1 peptide (B7, Table 2). One of these B19-restricted pools was also significantly positive in PBMC, the other B19- and the B4-pool did not induce a significantly positive response in spleen or PBMC (data not shown).

None of the B12 restricted pools 3, 4 and 12 induced a positive IFNγ response in MHC B4, B15, B19 or B21 birds (data not shown). Thus, screening of NP- and M1-derived peptide pools resulted in several candidate CD8+ T-cell epitopes that are restricted by different MHC types.

### Identification of AIV-specific CD8+ T-cell epitopes using individual peptides

**Figure 4:**
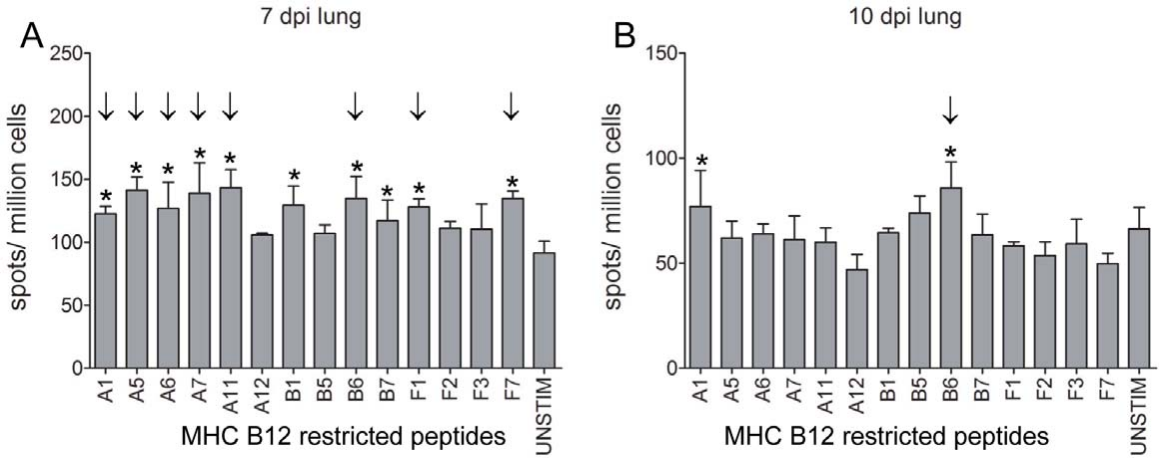
Screening of MHC B12-restricted CD8+ T-cell epitopes using individual peptides. Lung cells were isolated, *in vitro* re-stimulated with B12-restricted peptide pools and IFNγ-producing cells were determined by IFNγ ELIspot analysis at 7 dpi (A) and 10 dpi (B). Mean plus SEM is shown, n = 4 per group. Positive responses (*) and “significant” peptides inducing a positive response in 2 out of 3 chickens (↓) are indicated.

**Figure 5:**
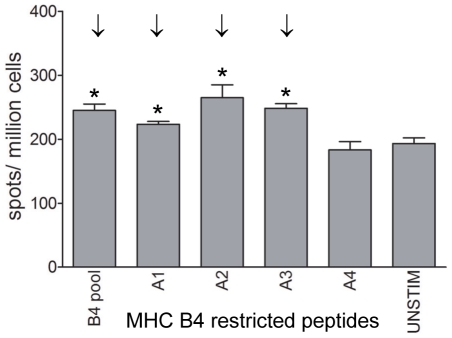
Identification of MHC B4-restricted CD8+ T-cell epitopes using individual peptides. Lung cells isolated at 10 dpi were thawed and re stimulated with B4 restricted peptides and IFNγ producing cells were determined by IFNγ Elispot analysis. Mean plus SEM is shown, n = 3 per group. Positive responses (*) and “significant” peptides inducing a positive response in 2 out of 3 chickens (↓) are indicated.

The initial screening of pools of predicted B12-restricted peptides resulted in 14 candidate epitopes. These peptides were tested individually together with a peptide from a pool that was not significantly positive in the first screening as a negative control. At 7 dpi, all 14 peptides induced IFNγ-producing cells in the lung, and 8 peptides were significantly positive (Fig. 4A). Also in PBMC many peptides tested significantly positive (Fig. S1C). Six peptides were both significantly positive in lung and PBMC (A1, A5, A6, A7, A11, F7, Table 1), 1 peptide was significantly positive in lung only (B6, Table 1) and 4 peptides were significantly positive only in PBMC (A12, B5, F2, F3, Table 1). However, these peptides induced IFNγ production in lung cells as well, but this was not significant. At 10 dpi, the numbers of spots were lower compared to 7 dpi both in the lung (Fig. 4B) and PBMC (Fig. S1D). Also the number of significantly positive peptides had decreased: 1 peptide was still positive in the lung while 5 peptides tested positive in PBMC. Again, the peptide that was recognized by CD8+ T cells in the lung was recognized also by CD8+ T cells in PBMC and the peptides found positive in PBMC did induce IFNγ-producing cells in the lung also but, based on our criteria, failed to test significantly positive. Individual testing of the B4-restricted candidate epitopes on frozen lung cells resulted in 3 significantly positive peptides (Fig. 5).

None of the peptides induced a positive response in cells isolated from uninfected chickens. This was shown both in lung cells (Fig. S2A) and PBMC (Fig. S2B). Interestingly, IFNγ production by unstimulated cells was much lower in cells from uninfected chickens compared to cells isolated from AIV infected chickens. IFNγ production in unstimulated lung cells varied between 4±1 spots/200,000 cells in uninfected chickens versus 18±2 spots/200,000 cells in AIV infected chickens (Fig. S3A). Similar results were observed in PBMC (Fig. S3B).

**Figure 6:**
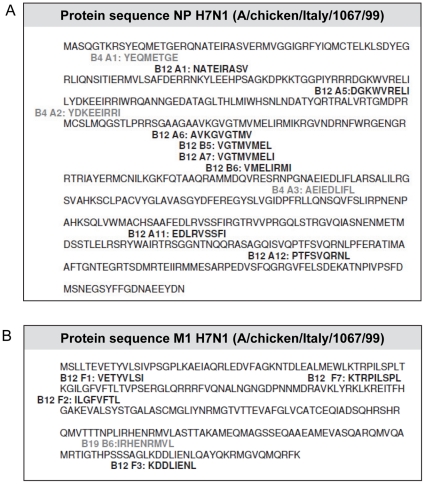
Novel AIV-specific CD8+ T cell epitopes within the LPAI H7N1 virus. Epitope mapping resulted in 11 novel CD8+ T-cell epitopes in the nucleoprotein (A) and 5 epitopes in the matrix 1 protein (B) of the LPAI H7N1 virus. In black, B12-restricted epitopes, in grey, B4-restricted epitopes (A) or B19-restricted epitopes (B).

Thus screening of AIV-specific CD8+ T-cell epitopes using lung cells of AIV-infected chickens resulted in 12 B12-restricted epitopes, 3 B4-restricted epitopes and 1 B19-restricted epitope. The novel AIV-specific CD8+ T-cell epitopes are distributed throughout the NP protein (Fig. 6A) and M1 protein (Fig. 6B) except for 4 B12 restricted epitopes (A6, A7, B5 and B12) which are overlapping. Different peptides from these regions are processed and presented by B12 molecules, suggesting that this region within the NP protein may be very immunogenic.

## Discussion

In the current study we set out to identify novel CD8+ T-cell epitopes in the NP and M1 protein of AIV. Based on epitope prediction, 33 epitopes restricted by 5 different MHC types were tested *ex vivo* using cells from MHC inbred chickens infected with a LPAIV H7N1. This resulted in the identification of 16 novel AIV-specific CD8+ T-cell epitopes; 12 B12-restricted epitopes, 3 B4-restricted epitopes and 1 B19-restricted epitope.

Most of the peptides induced IFNγ-producing cells at one or more time points post infection. Strict criteria were applied to select for positive peptides. A peptide was positive when it induced an IFNγ response that was higher than in unstimulated controls plus 2 times the standard error and positive in at least 2 out of 3 chickens. In this way only peptides that induced a response in the majority of chickens were selected, which is a prerequisite for the development of a cross-protective CD8+ T-cell vaccine in chickens. Screening of B12-resticted peptides resulted in 12 novel epitopes of which 7 were detected both in the lung and in PBMC. None of the peptides induced a response in uninfected chickens. Epitopes are randomly distributed within the NP and M1 protein except for an immunogenic region in NP that contains 4 overlapping epitopes. This region is similar to the human influenza virus-specific HLA-A2-restricted CD8+ T-cell epitope TMVMELVRMIK [35], except for position 7 where the valine (V) is replaced by an isoleucin (I). Within the M1 protein, the B12-restricted epitope F7 is homologous to the HLA-A2-restricted epitopes GILGFVFTL (M1_56–66_) and ILGFVFTLTV (M1_59–68_) [35], [36]. This suggests cross-reactivity between human and avian influenza viruses similar to what has been reported before [22]. Interestingly, all epitopes were rather conserved. For example, comparison to sequences of European H5N1 isolates resulted in 87.6–100% homology. Interestingly, more spontaneous IFNγ production was observed in cells isolated from AIV-infected chickens compared to cells isolated from uninfected chickens. This suggests a higher activation status of immune cells due to the presence of the influenza virus.

In contrast to the increase in numbers of CD8αα+ and CD8αβ T cells in lung and spleen no clear changes in percentages of total CD8+ T cells were observed between infected and uninfected birds or differences between lung and PBMC. The latter is in contrast with a study of De Bree et al [37] who reported a higher frequency of influenza-specific CD8+ T cells in lung compared to PBMC. However, in the current study frequencies of total CD3+CD8αα and CD3+CD8αβ cells rather than frequencies of antigen-specific CD8+ T cells were analysed by flowcytometry. Perhaps the frequencies of influenza-specific CD8+ T cells [38], [39] compared to those of the total CD8 T-cell pool are rather low, and we were not able to detect these differences. Studies with MHC class I tetramers are warranted to investigate differences in T-cell frequencies upon AIV infection in the chicken in more detail.

Influxes of CD8αα+ cells into the lung upon infection with LPAIV H7N1 and LPAIV H9N2 have been reported before [40], [41]. However, in these analyses no co-staining with CD3 was performed. Since chicken NK cells also express CD8α [42], these CD8αα+ cells most likely represent a population of NK cells rather than CD8αα+ T cells.

In humans and mice, influenza-specific CD8+ T cells are directed to a limited number of immunodominant epitopes [15], [35], [43]. Our results show several novel AIV-specific CD8+ T-cell epitopes, however dominant epitopes are lacking. This may be related to the organization of the chicken MHC complex. Chickens have a “minimal essential MHC” [32]; the properties of the single dominantly expressed class I molecules determine whether a chicken will respond to a particular pathogen like influenza. This is in sharp contrast to the huge and complex mammalian MHC that expresses multiple MHC class I molecules that present pathogen-derived peptides to CD8+ T cells. The combination of a dominant CD8+ T-cell epitope with a dominant MHC would be dangerous for the host; loss of the epitope, for example due to viral escape mechanisms, would result in loss of influenza-specific immunity and eventually death. The presence of multiple subdominant epitopes limits the risk of loss of CD8+ T-cell immunity in case of mutations in influenza-specific CD8+ T-cell epitopes.

In mammals, immunodominance is influenced by differences in antigen presentation and CD8+ T-cell repertoire [44], which are both affected by immunoproteasomes [45], [46]. The absence of these inducible proteasome genes in chickens [47] might provide an alternative explanation for the lack of immunodominant epitopes.

Due to the use of only predicted epitopes on selected proteins, we cannot exclude that part of the AIV-specific CD8+ T-cell response (either targeting other viral proteins or other epitopes within NP and M1 with non canonical binding motifs) were missed with this strategy.

In conclusion, we have identified novel AIV-specific CD8+ T-cell epitopes in chickens. This knowledge can be used to study the role of CD8+ T cells against AIV infection in more detail in this natural host for the influenza virus, and will be important for vaccine development. Since CD8+ T-cell inducing vaccines can be targeted towards viral proteins that are conserved between a large variety of influenza strains, such vaccines may provide better protection against newly arising virus strains than the current influenza vaccines.

## Materials and Methods

### Ethics statement

All animal experiments were performed in strict accordance to the Dutch Animal Experimentation Act and EU directives 86/609/CEE and 2010/63/EU related to the protection of vertebrate animals used for experimental and other scientific purposes. The experimental protocols were approved by the Committee on Animal Experiments of the University of Utrecht (DEC 2008.II.01.010) and performed in the Central laboratory Animal Research Facility of the University of Utrecht, which has AAALAC (Association for Assesment and Accreditation of Laboratory Animal care) accreditation.

### Animals

One-day old chickens of the following MHC inbred lines were obtained from Dr. H.J. Madsen: line 2 (MHC B12), line 4 (MHC B4), line 22 (MHC B15), line 21-19 (B19) and line 21-21 (MHC B21) [48]. Chickens were housed in groups and fed *ad libitum* on commercial feed. At 3 weeks, chickens were infected with the low pathogenic avian influenza strain H7N1 isolate A/chicken/Italy/1067/99 (kindly provided by Dr. W. Dundon). Virus was diluted in sterile PBS at a concentration of 1×10^8^ EID_50_/ml and chickens were inoculated intranasally and intratracheally (100 µl each). Uninfected controls chickens were inoculated with PBS. At 5, 7, 10 and 14 days post infection, birds were euthanized using cervical dislocation and lungs, spleen and blood were collected. In the second animal experiment, B12 birds were infected with H7N1 or PBS as described before. Birds were euthanized at 7 and 10 dpi and lungs and blood were collected.

### Isolation of cells

In order to obtain a single cell suspension, lung tissue was cut into small pieces and digested in RPMI containing collagenase A from Clostridium histolyticum and DNAse I isolated from bovine pancreas (Roche Applied Science, Almere, the Netherlands) for 30 min at 37°C, and homogenised using a 70 µM cell strainer (Beckton Dickinson (BD), Franklin Lakes, NJ, USA). Spleens were homogenised using a 70 µM cell strainer. Viable cells were isolated from lung, spleen and blood by Ficoll-Paque density gradient centrifugation. Cells were resuspended in “complete medium” which is RPMI medium supplemented with 10% heat inactivated FCS, 100 U/ml penicillin, 100 µg/ml streptomycin and 2 mM glutamax (Gibco BRL, United Kingdom) and were either used directly or frozen and stored in liquid nitrogen

### Peptide pools and peptides

The database SYFPEITHI (http://www.syfpeithi.de/home.htm) was used to predict epitopes in the AIV-proteins NP and M1 based on anchor residues described for MHC B4, B12, B19 and B21 [32]–[34]. Predictions resulted in 33 B12-restricted, 14 B19-restricted, 4 B4-restricted, 1 B15-restricted and 6 B21-restricted epitopes (Table 1, Table 2). Individual peptides of 8–11 amino acids were synthesized (Pepscan, Lelystad, The Netherlands), dissolved in DMSO, diluted in PBS to a concentration of 2 mg/ml and subsequently pooled. This resulted in 1 pool of B4-restricted peptides, 12 pools of B12-restricted peptides, 8 pools of B19-restricted peptides, and 1 pool of B21-restricted peptides. For the B12- and B19-restricted peptides, peptide pools were made using a matrix approach [49] to ensure that each peptide is part of two pools. B4- and B21-restricted peptides were pooled in one pool each (Tables S1 and S2). Peptide pools were aliquoted and stored at −20°C until use.

### Flowcytometry

Frequencies of CD8αα+CD3+, CD8αβ+CD3+, and CD4+CD3+ were analysed by flowcytometry. Cells were stained with mouse-anti-chicken CD3-PE (clone CT-1; IgG1) together with mouse-anti-chicken CD8α- FITC (clone CT-8; IgG1) and a biotin labelled mouse-anti-chicken CD8β (clone EP42, IgG2a). Alternatively, cells were stained with mouse-anti-chicken CD3-PE together with mouse-anti-chicken CD4-biotin (clone CT-4, IgG1) and mouse-anti-chicken TCRγδ (TCR-1, IgG1). All antibodies were obtained from Southern Biotech (SBA), San Diego, CA, USA. Stainings were performed for 20 min at 4°C. Next, cells were washed using PBS supplemented with 0.5% (v/v) bovine serum albumin (PBA) and stained with streptavidin-APC (BD Biosciences, Franklin Lakes, NJ, USA) for 20 min at 4°C. Cells were washed in PBA and fixed using a 2% final concentration of paraformaldehyde (Merck, Darmstadt, Germany) for 10 minutes at room temperature. Afterwards, cells were washed once in PBA and flowcytometry was performed. At least 50,000 events were acquired using a FACS Calibur flowcytometer (BD). All data were analyzed using the software program FlowJO (Threestar Inc, Ashland, OR, USA).

### IFNγ ELIspot analysis

IFNγ ELIspot was performed as previously described [50]. Briefly, 96 well Mulitiscreen® HTS, (Millipore, Billerica, MA, USA) were incubated with 70% ethanol for 1 min at room temperature and washed 1 time with H_2_O followed by 1 wash with PBS. Wells were coated with mouse-anti-chicken-IFNγ (2.5 µg/well in PBS; chicken IFNγ CytoSet™ (Invitrogen, Carlsbad, CA, USA) and incubated overnight at 4°C. Next, plates were washed 2 times with complete medium and blocked with complete medium at 41°C, 5% CO_2_. After 1 hour, complete medium was discarded and cells were seeded at 2×10^5^ cells/well in 200 µl in triplicate. Cells were stimulated with either peptides pools (final concentration of the individual peptides is 1 µg/ml) or individual peptides (1 µg/ml) for 24 hours at 41°C, 5% CO_2_. As a positive control for the capacity of cells to produce IFNγ a combination of 50 ng/ml phorbol myristrate acetate (PMA) and 500 ng/ml Ionomycin was used (Sigma-Aldrich, Zwijndrecht, the Netherlands). Unstimulated cells were used as negative control. Next, plates were washed 5 times with PBS supplemented with 0.05% Tween-20 (PBS-Tween) and incubated with anti-chicken IFNγ biotin (1 µg/well in PBS, Chicken IFNγ CytoSet™, Invitrogen) for 1 hour at room temperature. Plates were washed 5 times with PBS-Tween and incubated with poly-HRP (0.2 µg/well; Sanquin, Amsterdam, the Netherlands) for 1 hour at room temperature. Plates were washed again 5 times with PBS-Tween and TMB was added (50 µl/well, TMB for ELIspot, Sanquin). After spots became visible, plates were washed with tap water and airdried. Plates were analysed using the AELVIS automated spot analyzer (Sanquin). For a representative example, see Fig. S1A). Positive responses were defined as “the mean of the triplicates is higher than the mean of the triplicates plus 2 times the standard error of unstimulated control”. Peptides inducing a response in 2 out of 3 chickens were defined as “significant”.

## Supporting Information

Figure S1 **Screening of MHC B12-restricted CD8+ T-cell epitopes using individual peptides.** (A) A representative image of IFNγ Elispot results of lung cells isolated at 10 dpi and stimulated with medium (unstimulated), the B12 restricted peptide pool 3 or PMA/Ionomycin. Lung cells were isolated, *in vitro* re-stimulated with B12-restricted peptides and IFNγ-producing cells were determined by IFNγ ELIspot analysis. A representative example of lung cells isolated at 7 dpi is shown (B) together with results for PBMC isolated at 7 dpi (C) and 10 dpi (D). Mean plus SEM is shown, n = 4 per group. Positive responses (*) and “significant” peptides inducing a positive response in 2 out of 3 chickens (↓) are indicated.
(TIF)

Figure S2 **Screening of MHC B12-restricted CD8+ T-cell epitopes using individual peptides in uninfected birds.** Cells from uninfected (PBS infected) birds were isolated, *in vitro* re-stimulated with B12-restricted peptide pools and IFNγ-producing cells were determined by IFNγ ELIspot analysis at 7 dpi in lung (A) and PBMC (B). Mean plus SEM is shown, n = 3 per group.
(TIF)

Figure S3 **Higher spontaneous IFNγ production by unstimulated cells isolated from AIV-infected chickens.** Lung cells were isolated from uninfected and AIV-infected chickens, cultured for 24 hours *in vitro* in the absence of peptides and IFNγ-producing cells were determined by IFNγ ELIspot analysis at 7 dpi in lung (A) and PBMC (B). Mean plus SEM is shown, n = 3 per group. In white, responses in uninfected chickens; in grey, responses in AIV-infected chickens.
(TIF)

Table S1 **Thirty-three MHC B12 restricted individual peptides were assigned to 12 pools using a matrix approach.**
(DOC)

Table S2 **Fourteen MHC B19 restricted individual peptides were assigned to 8 pools using a matrix approach.**
(DOC)
