# Supplementary figures and images for: Identification of Novel Avian Influenza Virus Derived CD8+ T-Cell Epitopes
Source: PLoS One. 2012 Feb 23;7(2):e31953. doi: 10.1371/journal.pone.0031953 (PMC3285639; doi:10.1371/journal.pone.0031953)

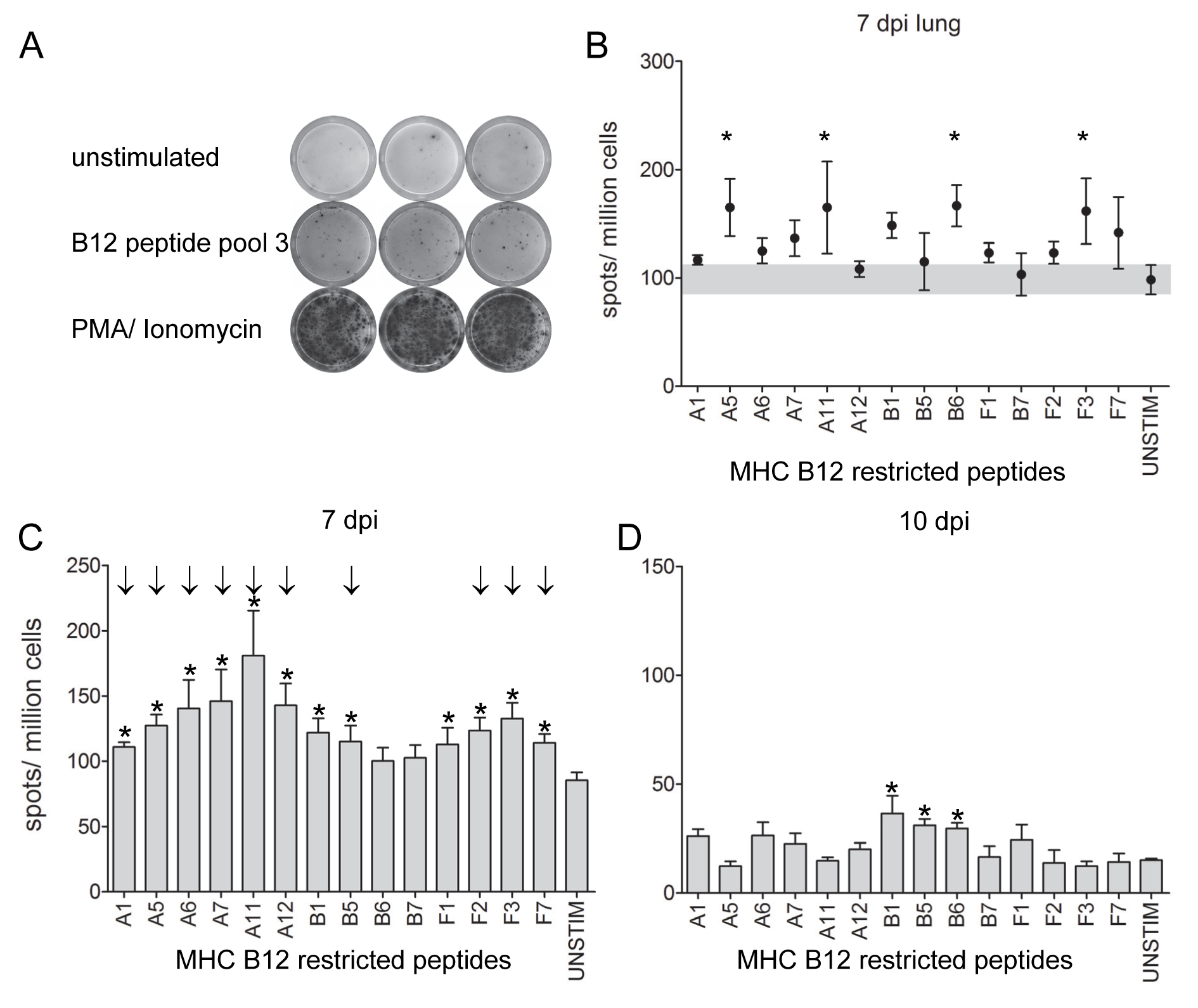

Supplement: Figure S1 — Screening of MHC B12-restricted CD8+ T-cell epitopes using individual peptides. (A) A representative image of IFNγ Elispot results of lung cells isolated at 10 dpi and stimulated with medium (unstimulated), the B12 restricted peptide pool 3 or PMA/Ionomycin. Lung cells were isolated, in vitro re-stimulated with B12-restricted peptides and IFNγ-producing cells were determined by IFNγ ELIspot analysis. A representative example of lung cells isolated at 7 dpi is shown (B) together with results for PBMC isolated at 7 dpi (C) and 10 dpi (D). Mean plus SEM is shown, n = 4 per group. Positive responses (*) and “significant” peptides inducing a positive response in 2 out of 3 chickens (↓) are indicated. (TIF) [file pone.0031953.s001.tif]

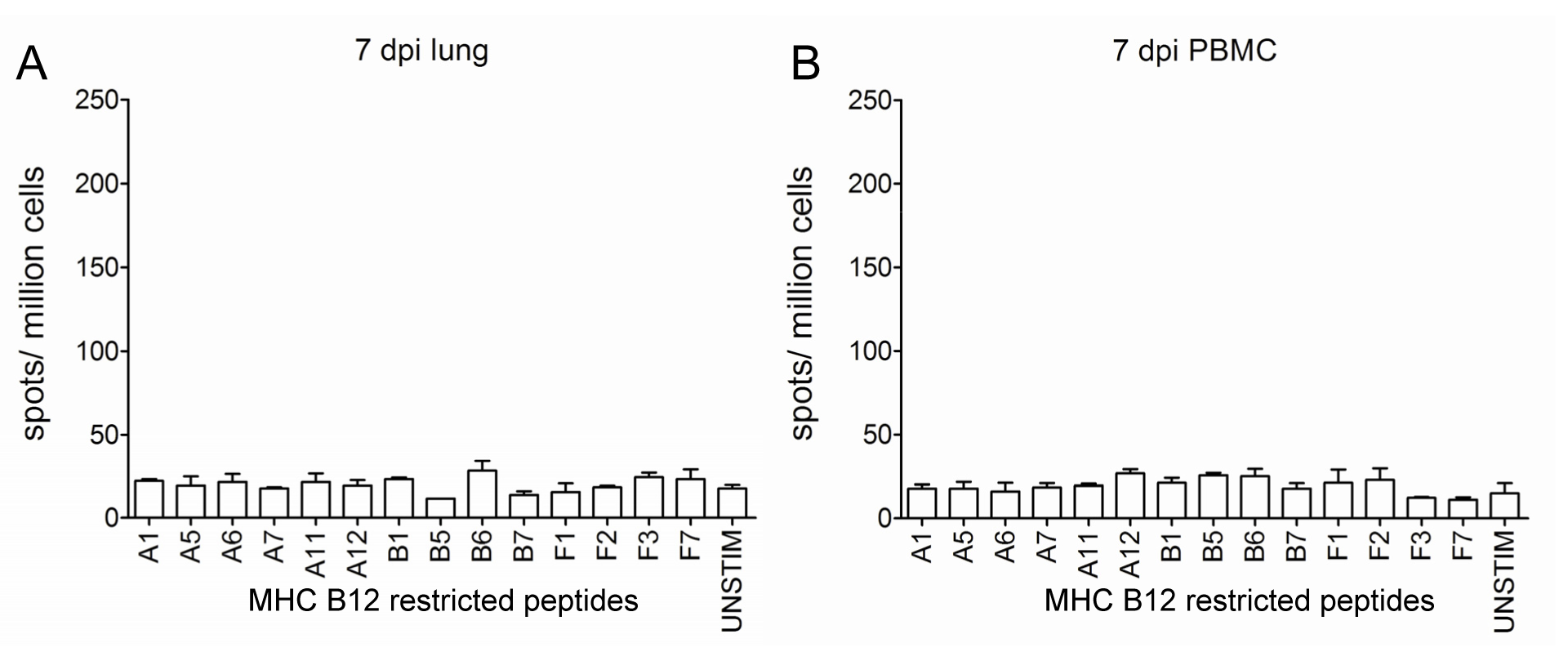

Supplement: Figure S2 — Screening of MHC B12-restricted CD8+ T-cell epitopes using individual peptides in uninfected birds. Cells from uninfected (PBS infected) birds were isolated, in vitro re-stimulated with B12-restricted peptide pools and IFNγ-producing cells were determined by IFNγ ELIspot analysis at 7 dpi in lung (A) and PBMC (B). Mean plus SEM is shown, n = 3 per group. (TIF) [file pone.0031953.s002.tif]

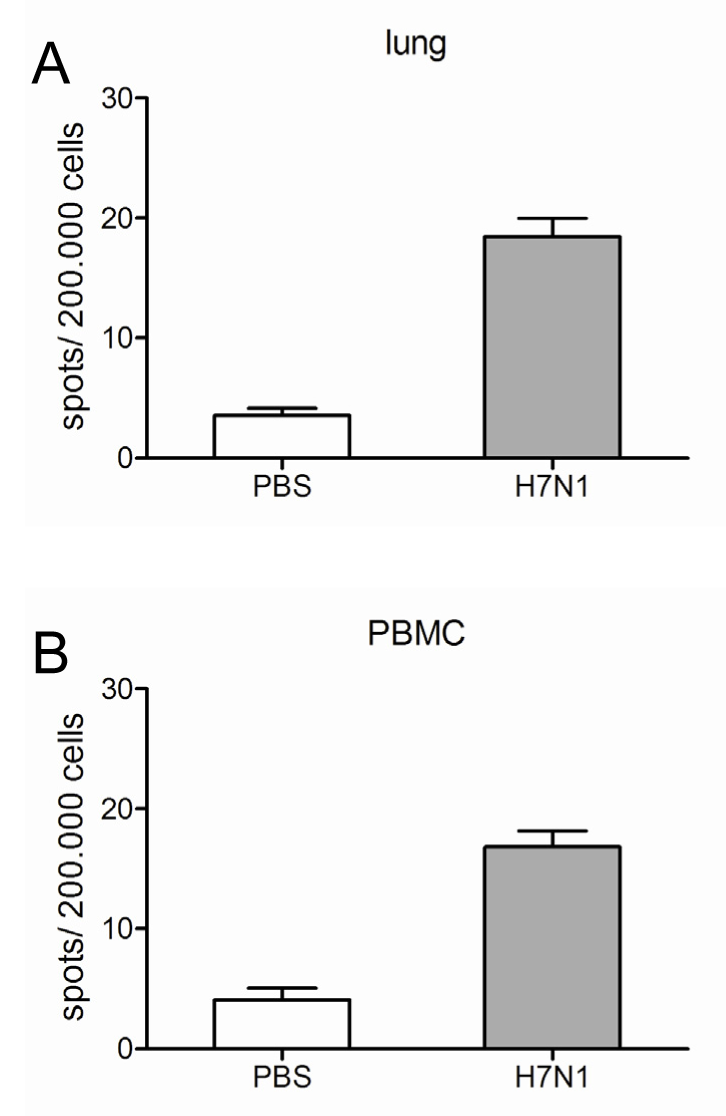

Supplement: Figure S3 — Higher spontaneous IFNγ production by unstimulated cells isolated from AIV-infected chickens. Lung cells were isolated from uninfected and AIV-infected chickens, cultured for 24 hours in vitro in the absence of peptides and IFNγ-producing cells were determined by IFNγ ELIspot analysis at 7 dpi in lung (A) and PBMC (B). Mean plus SEM is shown, n = 3 per group. In white, responses in uninfected chickens; in grey, responses in AIV-infected chickens. (TIF) [file pone.0031953.s003.tif]
