# Supplementary material for: Identification of Novel Avian Influenza Virus Derived CD8+ T-Cell Epitopes
Source: PLoS One. 2012 Feb 23;7(2):e31953. doi: 10.1371/journal.pone.0031953 (PMC3285639; doi:10.1371/journal.pone.0031953)
Supplement: Table S1 — Thirty-three MHC B12 restricted individual peptides were assigned to 12 pools using a matrix approach. (DOC) [file pone.0031953.s004.doc]

|  | Pool 7 | Pool 8 | Pool 9 | Pool 10 | Pool 11 | Pool 12 |
| --- | --- | --- | --- | --- | --- | --- |
| Pool 1 | A1 | A2 | A3 | A4 | A5 | A6 |
| Pool 2 | A7 | A8 | A9 | A10 | A11 | A12 |
| Pool 3 | B1 | B2 | B3 | B4 | B5 | B6 |
| Pool 4 | B7 | B8 | B9 | B10 | F1 | F2 |
| Pool 5 | F3 | F4 | F5 | F6 | F7 | F8 |
| Pool 6 | F9 | F10 | F11 |  |  |  |

Supplementary table 1. Thirty-three MHC B12 restricted individual peptides were assigned to 12 pools using a matrix approach.
