# Supplementary material for: Identification of Novel Avian Influenza Virus Derived CD8+ T-Cell Epitopes
Source: PLoS One. 2012 Feb 23;7(2):e31953. doi: 10.1371/journal.pone.0031953 (PMC3285639; doi:10.1371/journal.pone.0031953)
Supplement: Table S2 — Fourteen MHC B19 restricted individual peptides were assigned to 8 pools using a matrix approach. (DOC) [file pone.0031953.s005.doc]

Supplementary table 2. Fourteen MHC B19 restricted individual peptides were assigned to 8 pools using a matrix approach.

|  | Pool 19 | Pool 20 | Pool 21 | Pool 22 |
| --- | --- | --- | --- | --- |
| Pool 15 | A6 | A7 | A8 | A9 |
| Pool 16 | A10 | A11 | A12 | B1 |
| Pool 17 | B2 | B3 | B4 | B5 |
| Pool 18 | B6 | B7 |  |  |
